# Tissue Engineering Strategies for Treating Avascular Necrosis of the Femoral Head

**DOI:** 10.3390/bioengineering8120200

**Published:** 2021-12-02

**Authors:** Sumit Murab, Teresa Hawk, Alexander Snyder, Sydney Herold, Meghana Totapally, Patrick W. Whitlock

**Affiliations:** 1 Division of Pediatric Orthopaedic Surgery, Cincinnati Children’s Hospital Medical Center, Cincinnati, OH 45229, USA; hawktm@mail.uc.edu (T.H.); snydeag@mail.uc.edu (A.S.); sydney.herold@louisville.edu (S.H.); totapama@mail.uc.edu (M.T.); 2 Department of Orthopaedic Surgery, College of Medicine, University of Cincinnati, Cincinnati, OH 45267, USA; 3 Department of Biomedical Engineering, University of Cincinnati, Cincinnati, OH 45219, USA

**Keywords:** avascular necrosis, femoral head, osteonecrosis, tissue engineering, bone

## Abstract

Avascular necrosis (AVN) of the femoral head commonly leads to symptomatic osteoarthritis of the hip. In older patients, hip replacement is a viable option that restores the hip biomechanics and improves pain but in pediatric, adolescent, and young adult patients hip replacements impose significant activity limitations and the need for multiple revision surgeries with increasing risk of complication. Early detection of AVN requires a high level of suspicion as diagnostic techniques such as X-rays are not sensitive in the early stages of the disease. There are multiple etiologies that can lead to this disease. In the pediatric and adolescent population, trauma is a commonly recognized cause of AVN. The understanding of the pathophysiology of the disease is limited, adding to the challenge of devising a clinically effective treatment strategy. Surgical techniques to prevent progression of the disease and avoid total hip replacement include core decompression, vascular grafts, and use of bone-marrow derived stem cells with or without adjuncts, such as bisphosphonates and bone morphogenetic protein (BMP), all of which are partially effective only in the very early stages of the disease. Further, these strategies often only improve pain and range of motion in the short-term in some patients and do not predictably prevent progression of the disease. Tissue engineering strategies with the combined use of biomaterials, stem cells and growth factors offer a potential strategy to avoid metallic implants and surgery. Structural, bioactive biomaterial platforms could help in stabilizing the femoral head while inducing osteogenic differentiation to regenerate bone and provide angiogenic cues to concomitantly recover vasculature in the femoral head. Moreover, injectable systems that can be delivered using a minimal invasive procedure and provide mechanical support the collapsing femoral head could potentially alleviate the need for surgical interventions in the future. The present review describes the limitations of existing surgical methods and the recent advances in tissue engineering that are leading in the direction of a clinically effective, translational solution for AVN in future.

## 1. Introduction

### 1.1. Avascular Necrosis

**Figure 1:**
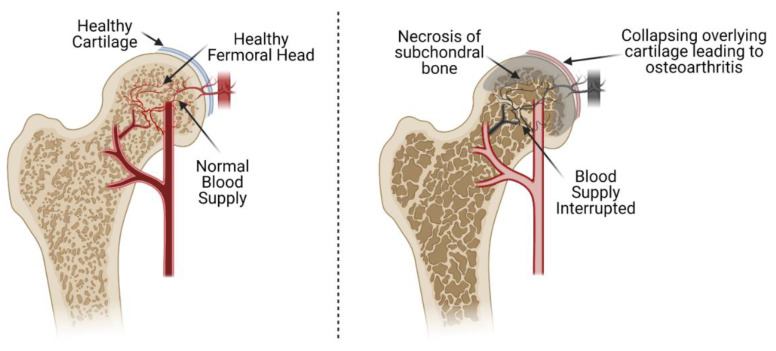
Avascular necrosis of the femoral head. The interruption of blood supply creates a hypoxic environment in the femoral head, leading to necrosis and collapse of the subchondral bone. This ultimately leads to collapse of the overlying cartilage of the femoral head and initiation of osteoarthritis of the femoral head and acetabulum.

Avascular necrosis (AVN) of the femoral head is caused by the interruption of blood supply to the bone of the proximal femur. The loss of blood supply can result from both traumatic and non-traumatic causes and leads to cell death (osteonecrosis). Significant necrosis leads to total collapse of the overlying articular cartilage of the femoral head secondary to failure of the underlying necrotic subchondral bone (Figure 1) [1]. This occurs before revascularization and regeneration of the necrotic bone can occur due to loss of the underlying structural rigidity of the affected bone as it is resorbed by osteoclasts. The end result of this process is early osteoarthritis (OA) of the hip. As the osteoarthritis progresses, the patient will often require total hip replacement (THA) [2]. In the USA, an estimated 20,000–30,000 cases are diagnosed with AVN annually, out of which 5–12% will need subsequent surgery [3]. Several factors can lead to the pathogenesis of AVN of the femoral head, but the exact pathophysiology of the disease has yet to be described. Non-surgical strategies have been described for the treatment of AVN, but surgical interventions remain the prominent therapeutic strategy.

### 1.2. Etiologies

Several different etiologies can result in the occurrence of AVN of the femoral head. The etiologies can be divided into traumatic and non-traumatic categories. In this review, we will focus our attention upon traumatic injuries as these are the most understood with regard to pathophysiology, have a high suspicion for the development of AVN and thus early recognition, and present a scenario where tissue engineering strategies might have the greatest impact and success.

Common causes of traumatic AVN include fracture of the femoral neck and femoral head dislocation from the acetabulum (hip dislocation). Both result in the disruption of blood supply (medial circumflex femoral artery) to the femoral head and its epiphysis in skeletally immature patients, causing the initiation of AVN. Trauma causes the retinacular and synovial vessels to detach, rupture or constrict as a consequence of fracture of the femoral neck as they are tethered to its surface. Destruction of retinacular vessels adjacent to the fracture line is proportionate to posterior comminution and displacement. Systemic induction of necrosis can be caused due to lesions in the epiphyseal as well as retinacular arteries that finally lead to the collapse of the femoral head [4]. Venous lesions, both in the form of thrombosis and rupture, can also lead to avascular necrosis. Rupture in the superior retinacular vessels makes the femur head reliant on the inferior retinacular and ligament teres vessels [5]. Sometimes, the displacement can be very severe and lead to lesions in the inferior retinacular vessels, which leaves just the ligament teres vessels for regeneration of the vasculature to the femur head [6]. However, a significant number of clinical and basic studies have shown that the ligament teres vessels are insufficient for the regeneration of vasculature to the femur head [4].

Apart from a direct vascular injury, several other factors increase risk for avascular necrosis. During low grade displacement, the integrity of the capsule may cause concentration of hematoma, resulting in increased pressure and an external tamponade of the intact vessels [7]. Similarly, an increase in the intracapsular pressure can have additional consequences such as arterial and venuous thrombosis, that can further lead to limited blood supply and thus increase the risk of necrosis of the femoral head or epiphysis [7]. Increased intra-articular pressure in femur neck fracture without displacement may result in limited blood supply and necrosis. It has also been reported that increased internal rotation of fracture of the femoral neck during fixation may increase the intra-capsular pressure. Lastly, iatrogenic injury to the vessels during surgery may also prodice AVN. Thus, maintenance of vascularization and the rapid initiation of revascularization are the key factors in impeding necrosis progression.

AVN will occur in 15–50% of cases of femoral neck and head fractures and 10–25% of hip dislocations [8]. Internal fixation is generally preferred in young patients in order to save the natural femoral head after fracture, while in older patients and elderly patients primary arthroplasty may be performed in order to restore mobility. Even after internal fixation, AVN of the femoral head is a common complication. Even a non-displaced femoral neck fracture treated with percutaneous pinning and closed reduction can result in AVN, after which 10% cases may lead to THA after 5 years [9]. AVN can also be caused in the pediatric population by an antegrade-intramedullary nailing through a piriformis entry point. Most of the patients who develop AVN report disabling pain, decreased function of the hip, with subsequent collapse of the femoral head and overlying cartilage leading to secondary osteoarthritis [10]. Procedures such as vascular fibular grafting and core decompression that have demonstrated some efficacy in preventing collapse of the femoral head, are more successful in the early stages of AVN [11]. Therefore, suspicion of AVN in its early stages is critical for the success of current surgical approaches to the treatment of AVN [12].

Lastly, common non-traumatic etiologies in the adult population include thrombosis due to corticosteroid abuse, alcohol abuse and radiation therapy. In the pediatric population, malignancy, chemotherapy, steroid use and sickle cell associated AVN are commonly encountered.

### 1.3. Pathophysiology of AVN

Necrosis of bone tissue occurs similarly in adults and children, but the femoral head differs significantly in cartilage maturity. In children, the epiphysis and proximal femoral physis are active and the chances of regeneration of the affected bone are theoretically higher when compared to the adults who have completed growth. Though pathophysiology of the disease is poorly understood, its phases have been described in general, phenomenologic terms. Bone necrosis happens in two phases. The first is ischemia which is followed by regeneration [13].

#### 1.3.1. Ischemia

As AVN may go unrecognized prior to becoming symptomatic, the exact period of the onset of ischemiahas not been clearly defined. The specific etiology both in children and adults may also be hard to identify in some cases. In pediatric non-traumatic cases of AVN, the etiology remains poorly understood and several theories were developed in the last century, including disruption of vessels that supply blood to epiphysis, thrombosis and cartilage piercing vessels that have been supported by available pathological data [13,14,15]. The constitutional theory suggests that the abnormal growth of cartilage tissue leads to the destruction of blood supply to the epiphysis, which will result in ischemia of the femur head [16]. Regardless, both adult and pediatric AVN patients most commonly seek treatment well after the pathology has passed the regeneration phase, indicating that the ischemic phase is minimally symptomatic.

As initial ischemia is not linked with any changes in the mineral content of the bone tissue, X-ray images are not sensitive to initial changes. Bone scintigraphy is sensitive for AVN and will show decreased blood flow in the femur head due to decreased blood supply [17,18]. MRI is similarly helpful in detecting the early changes in bone marrow due to ischemia and is the current standard of care for the diagnosis of AVN [19]. Only when the necrotic bone goes into the regeneration phase and mesenchymal stem cells (MSCs) are recruited to regenerate bone, is plain film X-ray imaging able to detect the progression of AVN. Thus, X-ray imaging is only useful if the pathology has already progressed into the regeneration phase [20]. The initial clinical diagnosis of AVN is performed with a careful history, physical exam and then imaging, but final, definitive confirmation is carried out with the histological results from a tissue biopsy. Imaging of the histological slides with a light microscope helps in detecting the dying cellular population of the tissue that includes bone marrow cells followed by osteoblasts and osteocytes in order of death due to necrosis. Histology is the gold standard to define and confirm tissue necrosis. One of the defining histological hallmarks of AVN is the disappearance of osteocytes from bone lacunae, though this condition may take up to 4 years in some patients [21]. Advanced pathological tests such as scanning electron microscopy and DNA visualization assays have shown that the osteocytes might be present inside the bone lacunae but are mostly non-viable [13]. While it is widely reported that the ischemic phase in the pathology of AVN is similar in both adults and children with respect to interruption of the arterial blood supply, there are some reports that describe the heritable thrombosis causing venous occlusion and subsequent bone necrosis due to venous hypertension [22]. An important sign is the reduction in the population of the endothelial progenitor cells that results in the lack of neo-angiogenesis in the necrotic femoral head during the progression of AVN [23].

#### 1.3.2. Regeneration

After the blood supply to the femoral head is disrupted and the necrosis begins, the molecular cues for bone healing help in recruiting the MSCs to the necrotic site [24]. This is initiated by the formation of cartilage piercing blood vessels growing in the same direction as the medial circumflex artery [25]. This pathological signature can also be utilized for some therapies [26]. Prior studies have demonstrated excessive revascularization before the onset of ischemia and abnormal cartilage growth caused the femoral head to grow bigger that the acetabulum, which again placed the femoral head cartilage at increased risk [27]. This excessive neo-vascularization brings in both MSCs and monocytes that help in bone remodeling. Two opposing processes begin simultaneously in this phase [6]. The outer subchondral bone starts becoming resorbed by the active multinucleated osteoclasts formed from monocytes, while the core is acquiring more tissue constructed due to the anabolic activity of the osteoblasts. This degeneration of the subchondral bone can be observed in both adults and children through X-ray imaging and is described as a subchondral fracture line [28]. The degenerating subchondral bone results in the collapse of the overlying articular cartilage. The cartilage is not particularly affected in AVN pathology but is affected due to collapse of the supporting subchondral bone to which it is anchored and depends [29]. In adults this will finally result in hip osteoarthritis, while in children it depends on the regeneration capacity of the epiphyseal cartilage and can result both in the destruction of joint or its at least partial restoration and continued function. The regeneration depends on the age group of the child, below 4 years generally have a full recovery, while regeneration of the femoral head after 4 years of age becomes much less predictable. The ability to restore the original shape and height of the femoral head is called biological plasticity. In order for the femoral head to recover, it needs to be fully covered in the acetabulum and the motion should be maintained, which can be helped with surgical interventions and is called containment treatment [13]. It has also been found that revascularization of the necrotic femoral head can been observed under scintigraphy, but the same can also be observed in cases where necrosis has not yet begun. Thus, while AVN can be silent and be difficult to diagnose in the early stages on plain films, this phenomena allows it to be diagnosed by scintigraphy or MRI. The vascularization of subchondral bone is followed by formation of fibrous tissue in place of the degenerating bone tissue, which results in the collapse of the femoral head. It is followed by flattening of the head and injuries to the overlying cartilage and subsequent development of osteoarthritis [30]. Further, excessive revascularization may also lead to increased anabolic activity, resulting in the formation of an endochondral callus, if one of the fracture cleft surfaces is viable [31].

### 1.4. Cell and Tissue Necrosis

**Figure 2:**
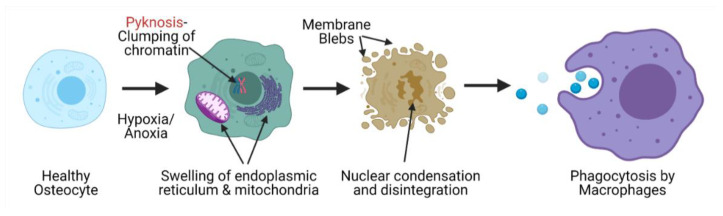
Mechanism of cell necrosis during avascular necrosis of the femoral head that leads to the clearance of osteocytes from bone lacunae, a confirmatory histological signature of avascular necrosis.

AVN involves an organized pattern of cellular necrosis and a complicated progression of bone tissue formation and resorption [32]. The cellular pathology of AVN includes the initial necrosis of adipocytes and hematopoietic cells. This is followed by the edema of the interstitial marrow. It is reported that osteocytes start necrosis as soon as 2–3 h from the beginning of oxygen exhaustion due to destruction of blood supply, though histological signatures of this can only be observed after 24–72 h after the initiation of nuclear pyknosis and occurrence of empty bone lacunae. Pyknosis is the irreversible condensation of the chromatin network in a necrotic cell, which is later followed by nuclear fragmentation. Apart from the nuclear changes, the cellular organelles begin to swell and rupture and are finally removed through phagocytosis (Figure 2) [33]. Necrosis of the bone tissue is followed by repair responses that involves capillary revascularization and reactive hyperemia around the necrotic tissue, thus initiating both bone resorption and neo-osteogenesis in order to remodel the dead tissue. The new bone tissue is layered over the dead trabeculae with partial bone resorption. The destruction of subchondral bone is a result of increased bone resorption as compared to the regeneration that results into weakening of bone trabeculae in this region followed by subchondral fracture and joint collapse. This pathology is due to tissue necrosis but primarily, is due to the imbalance between bone formation and resorption triggered by necrosis [34]. An in-silico study using finite element modelling demonstrated that the subchondral fracture is a result of the decreased integrity of the cancellous subchondral bone trabeculae as compared to the subchondral plate [35]. These tissue changes can be observed in X-ray images in which lucency is consistent with areas of increased bone resorption, while areas of sclerosis demonstrate trabeculae that can be either dead or under repair [36]. To develop treatment therapies that can help regenerate the necrotic femoral head, a better understanding of the cellular and molecular pathobiology of AVN is needed. A better understanding of the complex interplay at work during AVN, will help us define molecular targets that play crucial roles in the initiation, progression or healing after AVN, which may then be inhibited or accelerated to help halt the disease.

### 1.5. Disadvantages of Surgical Strategies

**Figure 3:**
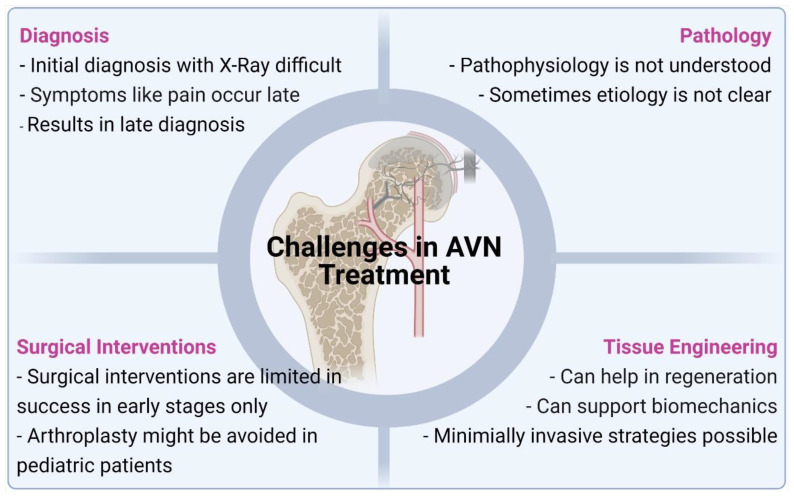
Common reasons for the limited success of currently available treatments of AVN and the advantages that future tissue engineering based strategies may offer.

The current treatment strategies for managing AVN mainly involve surgery. In both the adult and pediatric populations, surgical strategies used for the management of AVN are unpreditable and associated with high failure rates. Further, arthroplasty for osteoarthritis following AVN, poses significant restrictions and need for multiple revision surgeries during the patient’s lifetime. Multiple revisions of total hip arthroplasty in both young and old patients are also associated with increased chances of infection and other complications apart from decreased quality of life for these patients. Pharmacological agents have also been shown to be poorly effective in adult and pediatric patients as the pathophysiology of the disease is still sparsely understood. Currently, there are no targeted molecules or therapy available for the management of the disease. Due to unsatisfactory results with the use of pharmacological agents and surgical interventions (Figure 3) of AVN of the femoral head, especially in later stages of the disease, focus has been shifted to tissue engineering strategies to provide better treatment options and clinical outcomes for patients. Tissue engineering or regenerative medicine strategies can potentially help in regenerating the vasculature to the femoral head, which is the main cause of the disease while also helping in the regeneration of the necrotic bone tissue. Another area where the tissue engineering strategies need to focus is on the preservation of the initial biomechanical stability of the collapsing femoral head, while bio-inductive factors are recruited for initiating the regeneration of the vascular and bone tissue. Injectable therapies that would provide these cues and maintain biomechanical stability to prevent articular collapse and osteoarthritis while revascularization and bone regeneration can occur would be a potential treatment strategy for AVN in the pediatric population.

## 2. Desired Characteristics of 3D Scaffolds

Tissue engineered 3D scaffolds for bone tissue engineering have been extensively studied [37]. These 3D scaffolds can be utilized to provide mechanical support to collapsing femoral heads while delivering biochemical and biophysical cues for the induction, proliferation, and differentiation of MSCs for bone regeneration. Scaffolds can also be helpful in inducing vascularization in the necrotic tissue that can enhance the bone regeneration and remodeling process. 3D scaffolds can be injected or surgically implanted into patients’ femoral head, depending on the type of scaffold. Many types of materials have been successfully used as 3D scaffolds, including natural metals and 3D printed synthetic materials. Since scaffolds have such a complex role in bone tissue regeneration, certain characteristics ensure their highest success: biocompatibility, degradability, porosity, mechanical performance, effect of growth factors (GF), effect of cell combinations, and in vivo regeneration capacity.

### 2.1. Biocompatibility and Degradability

Scaffold biocompatibility is defined as the ability of a scaffold to promote normal cellular functioning around its implanted location, without creating adverse reactions, such as cytotoxicity, immunogenicity, and swelling. Biocompatibility is necessary for the efficient induction and adhesion of stem cells or other osteoinductive cells. Several 3D scaffolds have been tested and observed to have good biocompatibility, such as polylactic acid (PLA), poly lactide-co-glycolide (PLGA), alginate, and tantalum metal. Degradability is another important characteristic for scaffolds; ideally as the bone tissue is regenerating, the scaffold should dissolve to make room for native regenerated tissue. Good degradability involves easy release of stem cells or drugs housed within the scaffolds and simple breakdown of the scaffold itself. Given that osteonecrosis often occurs in the femoral head, degradation and subsequent regeneration and sustained performance of the novel tissue is extremely important. The requirement for surgical removal of grafts or scaffolds from the hip has the potential to cause damage and adverse effects in a patient. Similar to biocompatibility, scaffolds must not create an immune response during degradation due to harmful chemicals. Several materials used in 3D scaffolds, such as polycaprolactone (PCL), alginate, and PLGA, have been tested with good biocompatibility and degradability [38]. Bioactivity is another property that is useful when inducing cells to behave in a specific manner. It may be the result of surface chemistry, shape, surface roughness, porosity or the inclusion of bioinductive materials for release form the scaffold itself. 

Many scaffold materials have properties which perform well in one categories described above, while performing undesirably in others. Thus, previous studies have attempted to use a combination of materials for fabricating tissue engineering scaffolds, which incorporate the desired properties from each material within the scaffold. Lai et al. aimed to capitalize on the benefits of multiple-material scaffolds. They used magnesium incorporated into poly lactide-co-glycolide/ß-tricalcium phosphate (PLGA/TCP) porous scaffolds in the rabbit model which showed good biocompatibility. They saw an increase in bone formation and strengthening of new bone, without any negative side effects, such as an immune response [39]. The scaffolds also decreased in volume over 12 weeks, showing good degradability in vivo. The addition of magnesium into the scaffolds allowed for improved biocompatibility and degradation over the standard PLGA/TCP scaffold. Similarly, Qin et al. used a novel PLGA/TCP scaffold with icaritin as a bone filler for prevention of femoral head collapse in the emu model. They found that the scaffold promoted MSC migration to the implant location, all while preventing undesired cell differentiation. The addition of icaritin to the scaffolds allowed increased calcium deposition and expression of osteogenic genes. The icaritin-containing scaffold also displayed no decrease in degradability [40]. Deng et al. used a porous selenium and silicon dioxide nanocomposite scaffold in the rat model over 8 weeks. They observed good biocompatibility with no inflammatory response. The scaffolds were able to reduce oxidative stress, thereby providing a method to protect bone from steroid induced osteonecrosis [41]. Other studies have shown that trans-cinnamaldehyde (TCA) has anti-inflammatory effects in vitro and can inhibit cartilage destruction [42]. Gao et al. used TCA to create a porous titanium alloy scaffold and observed it in a canine model over 12 weeks. They saw an increase in proliferation and differentiation of osteoblasts, while minimizing the inflammatory response [43]. Kawai et al. created a functionally graded PCL/b-TCP scaffold and implanted it into the rabbit model to observe biocompatibility and degradation after 8 weeks. The scaffold showed good biocompatibility with more bone ingrowth. The 3D scaffold had a sufficient degradation rate in vivo, as well [44]. Shen et al. showed that a novel polycaprolactone-polyethylene glycol-polycaprolactone (PCL-PEG-PCL) and MgO scaffold had increased cytocompatibility with MC3T3-E1 pre-osteoblast cells in vitro and in the rat model over 8 weeks [45]. They demonstrated high cell viability on the scaffold along with increased proliferation and differentiation. The scaffold also showed a good degradability profile due to the addition of poly ethylene glycol (PEG) component. Moreover, the study saw that low magnesium oxide concentration was favored both in vitro and in vivo in the rat model. Another study observed that a novel 3D PCL scaffold promoted angiogenesis and osteogenesis, both in vitro and in vivo. In vitro, the scaffold increased vascularization in human umbilical endothelial cells, enhanced mineral matrix production, and increased expression of osteogenic genes. The scaffold also showed great biocompatibility through attachment and differentiation of BMSCs. Moreover, the scaffold showed good degradability in vivo [46]. Finally, Guillaume et al. demonstrated that a poly-trimethylene carbonate scaffold with hydroxyapatite particles and BMSCs promoted osteogenic differentiation in the rabbit model over 6 weeks [47]. They saw significant bone regeneration, cell proliferation, and cell attachment to the scaffold. The scaffold also showed good biocompatibility and degradability due to the polycarbonate polymer. This study offers an interesting example of a 3D scaffold, as it does not use a porous metal to achieve promising results. Just as all of these studies describe, the combination of multiple materials can have significant additive effects on scaffold properties. Addition of bioactive or bio-inductive materials such as growth factors, minerals, or microspheres, therefore, may maximize both biocompatibility and degradability in producing an optimal scaffold for bone and vascular regeneration after avascular necrosis.

### 2.2. Porosity

Another important characteristic of scaffolds that can affect the rate of tissue regeneration is their porosity, which is the amount of empty space within the scaffold. 3D scaffolds aim to be similar to natural bone tissue, of which porosity is an important component. Bone tissue has a specific pore structure and porosity that allows proper growth, strength, and proliferation. One factor that complicates scaffold creation is that cancellous and compact bone have widely different porosities. This means that the type of bone the scaffold will be present in must be taken into account. Porosity is able to increase osteogenesis by promoting vascularization and allowing migration of stem cells. This is particularly important for osteonecrosis, where the lack of blood circulation prevents osteogenesis. Thus, the porosity of a scaffold can directly affect its ability to improve osteonecrosis. Porosity can also enhance stem cell attachment via an increase in surface area and roughness leading to enhanced osteogenesis. However, there is a limit on the porosity of 3D scaffolds. Structural strength and mechanical performance have to be balanced with porosity in order to maintain optimum functioning in vivo. Additionally, degradation should be taken into account when selecting the porosity of a scaffold. Scaffolds that have a high degradation rate should not have high porosity. This is due to the fact that accelerated degradation will compromise the structural integrity of the scaffold. The opposite is also true: scaffolds with a low degradation rate should be highly porous to maximize osteogenic ability. Torres-Sanchez et al. observed that the best 3D scaffolds have pore sizes of less than 212 µm and 27–37% porosity for compact bone in a 12-day in vitro study using porous titanium scaffolds [48]. The study also found that scaffolds for trabecular bone had optimum measurements of 300–500 µm and 54–58% porosity. These measurements provided the maximum amount of cell adhesion and proliferation with osteosarcoma osteoblasts. Additionally, pore sizes >300 mm showed the most cell proliferation, while pore sizes between 45 and 106 mm showed the greatest cell adhesion. Grier et al. observed that equine tenocytes had higher proliferation, higher metabolic activity, and increased expression of genes such as COL1A2, COMP, and DCN on scaffolds of smaller pore size and higher crosslinking densities [49]. The study used collagen-GAG scaffolds and observed the results in vitro over 14 days. They saw a trend that smaller pore size corresponded to higher cell proliferation and increased gene expression. One study used PLGA/TCP porous scaffolds in the rabbit model and saw an increase in bone formation [39]. Their scaffold showed the great similarity to human trabecular bone and had 80% porosity and 400 mm pore size. The addition of porous magnesium into the scaffold created an environment more suitable for osteogenesis. Similarly, Xiao et al. found that porous b-TCP scaffolds with an interconnection size of 150 mm had the highest incidence of angiogenesis, human umbilical vein endothelial cell proliferation, and cell migration in a rabbit femoral defect model [50]. In an interesting example of how nature can influence bioengineering, several studies have used biomimetic scaffolds in vitro that are similar to the structure of lotus flower. Feng et al. used a silicate-based akermanite scaffold similar to the lotus root with increased porosity over traditional scaffolds and showed higher BMSC attachment and proliferation. They also saw improved osteogenesis and angiogenesis due to the highly channeled structure [51]. Additionally, another study found that using a highly porous b-TCP scaffold with a structure similar to the lotus seed pod was able to significantly promote angiogenesis, molecular release, BMSC differentiation. It also showed good biocompatibility in the rat model [52]. Rnjak-Kovacina et al. used porous silk scaffolds with arrayed hollow channels with human dermal neonatal fibroblasts in vitro. They demonstrated that the addition of porosity through arrayed hollow channels allowed cell infiltration, host integration, and vascularization. The study further explained that this technique could then be used for large tissue formation [53]. Similarly, another study created a novel PCL-PEG-PCL scaffold incorporated with porous magnesium. They found that the scaffold had porosity similar to cancellous bone and showed increased cell adhesion, proliferation, and differentiation [45]. Finally, Yan et al. used PCL scaffolds with deferoxamine and saw increased angiogenesis and osteogenesis. The scaffold had a porosity of approximately 39%, successfully released the deferoxamine, and showed good BMSC attachment. Additionally, they adjusted the porosity of the scaffold to maximize its osteogenic capability and increase its similarity to cancellous bone. Using the 3D scaffold, Yan et al. were able to promote bone growth and osseointegration in a bone defect rat model [46]. Thus, porosity plays a crucial role in designing scaffolds specifically for bone regeneration in order to mimic and facilitate the formation of bone. It also plays a key role in the vascularization of tissue engineering constructs, as the new vessels need optimum porosity to grow inside these scaffolds for optimum nutrient transportation as well as vasculature repair.

### 2.3. Mechanical Performance

While porosity promotes cell attachment and bone ingrowth, there are limits as to how porous a 3D scaffold can be. As previously stated, porosity increases the amount of empty space inside a scaffold, thereby decreasing its structural strength. Mechanical strength is extremely important in scaffolds, as the skeleton provides structure and load bearing ability for the rest of the body. This strength is determined by the inherent properties of the 3D scaffold material and its pore structure. Extreme porosity in a scaffold may lead to its failure in vivo when subjected to mechanical stress. Since osteonecrosis commonly occurs in the weight-bearing femoral head, mechanical performance is highly important. Therefore, balancing porosity with mechanical strength is a challenge when designing a 3D scaffold. 3D scaffolds must be able to withstand dynamic, physiologic compressive and shear loads at their site of implantation implantation site. They must also be able to support the implantation site long enough for the formation of new bone to occur. Scaffolds made of synthetic polymers mixed with natural inorganic materials commonly have high mechanical performance. Lai et al. used this method when they created a novel scaffold of PLGA/TCP combined with porous magnesium (PTM). Their scaffold showed a good biomimetic structure along with enhanced mechanical properties. The scaffold displayed higher compressive strength compared to the control without magnesium. Additionally, the PTM had a Young’s modulus very similar to that of trabecular bone. Furthermore, the scaffold still maintained increased osteogenesis and angiogenesis, showing that the desired porosity and osteoinduction could be maintained [39]. Similarly, another study found that icaritin incorporated into PLGA/TCP scaffolds had lower rates of femoral head collapse and promising mechanical properties in the rabbit model. The scaffold not only had higher rates of osteogenesis, but could also withstand higher compressive loads and give more stability to the implant location. The 3D scaffold had a maximum strength of about 47 Newtons and was about to hold approximately 0.02 joules of energy [40]. Kawai et al. used a functionally graded PCL/b-TCP scaffold in the rabbit model and observed its mechanical properties. Both proximal and middle sections of the scaffold were within the compressive strength range of trabecular bone. Additionally, the 3D scaffold showed promising load bearing qualities for implantation [44]. Cui et al. found that a porous methacrylated glycol chitosan–montmorillonite hydrogel had enhanced mechanical properties suitable for tissue engineering. The scaffold also promoted cell infiltration, proliferation, and differentiation in a mouse calvarial defect model [54]. Chang et al. used bioceramic porous scaffolds combined with CaCO_3_ and observed a good compressive strength of 47 MPa. Additionally, the scaffold showed good biocompatibility with no cytotoxicity [55]. Shen et al. used a PCL-PEG-PCL scaffold combined with magnesium oxide to induce bone defect repair. The addition of MgO to the synthetic scaffolds significantly improved compressive strength and elastic modulus. The MgO combined scaffold had a compressive strength 23% higher than just the PCL-PEG-PCL scaffold alone. Moreover, this scaffold had compressive strengths within the normal range of cancellous bone [45]. These studies exemplify how the addition of natural molecules to synthetic scaffolds can enhance mechanical performance and maintain osteogenic capabilities. Other studies have used porous metal scaffolds to promote osteogenesis and angiogenesis, while retaining favorable mechanical performance. Torres-Sanchez et al. determined the optimum porosity and mechanical performance of a porous titanium scaffold in vitro. The study also demonstrated the relationship between scaffold porosity and mechanical strength in their metallic scaffold. They found that there was a power law correlation, where mechanical performance consistently decreased as porosity increased. The group’s novel scaffold also had an elastic modulus similar to that of cortical and trabecular bone but had a compressive strength 84% higher than that found in the bone types [48]. Similarly, Khodaei et al. used a porous titanium scaffold under different thermal oxidation conditions to assess its mechanical properties. They found that the scaffold had favorable mechanical properties in vitro. However, its compressive strengths decreased by almost 23% when thermal oxidation time was increased over 240 min. At optimum conditions, the scaffold showed a plateau stress value of about 51 MPa. Additionally, the scaffold showed good apatite formation under the optimum condition [56]. Overall, both combinations of natural and synthetic materials along with porous metallic scaffolds have shown promising mechanical properties for bone defect repair.

### 2.4. Effect of Growth Factors and Other Small Molecules

Just as 3D scaffolds can be improved by changing their material, they can also be enhanced by loading them with growth factors or other small molecules. 3D scaffolds have the ability to release growth factors or other molecules when introduced into a model. Growth factors (GF) can both stimulate osteoinductive stem cell differentiation and angiogenesis in vivo. Additionally, GFs can enhance cell proliferation and bone regeneration, further assisting in the repair repair of a bone defect. These are all extremely important in the setting of osteonecrosis since any treatment that can accelerate osteogenesis or angiogenesis could potentially improve patient prognosis and quality of life. Several growth factors are commonly used in conjunction with 3D scaffolds, such as vascular endothelial growth factors (VEGF), transforming growth factor b (TGF-b), and fibroblast growth factors (bFGF). Additionally, several types of bone morphogenic proteins (BMP), which are types of TGFs, have been used in scaffolds. Scaffolds can also be utilized to provide a reservoir for growth factors and other inducing molecules and ensure their sustained release in the implant region. BMPs are among the most effective growth factors in inducing osteogenesis and cell differentiation. BMPs also have the added benefit of angiogenesis when introduced into the body. BMP-2 remains the most common of the BMP family to be used in bone regeneration studies. Lin et al. used human BMSCs with BMP-2 in a hydrogel scaffold and observed the effects in severe combined immunodeficiency mice over 56 days. They saw long term BMP-2 production and increased rapid bone formation in the mouse model. In addition, the trabecular bone structure formed contained favorable vascularization for bone regeneration [57]. This study further shows how scaffolds can serve as a reservoir for GFs and prolong their release in the body. Similarly, Xia et al. used BMP-2 in a PLGA/gelatin microsphere scaffold in the rabbit model over 12 weeks. They found that BMP-2 scaffolds increased osteogenesis and proliferation of BMSCs in vitro. In vivo, BMP-2 scaffolds showed significantly higher osteogenic potential and sustained release of the GF [58]. Another GF commonly used in tissue engineering is VEGF. VEGF is a major component in regulating angiogenesis during bone regeneration. It can stimulate osteoblast proliferation along with vascularization in a bone defect region. Ren et al. used a polylactide-polyethylene glycol-polylactide microsphere scaffold with VEGF and rat MSCs to observe its osteogenic effects. They saw adequate GF release from the scaffolds in vitro over 46 days. Osteoblast differentiation was also significantly increased with the scaffolds. These results highlight the potential for use of this method for treatment of further bone defects [59]. Similarly, another study used hydroxyapatite collagen scaffolds with VEGF and observed GF release in vitro and in vivo in the rat model. They observed sufficient release of the GF in vitro. Additionally, the scaffolds showed osteoblast differentiation of the stem cells along with induced osteogenesis in vivo. They also observed vascularization that they concluded resulted from VEGF and the scaffold [60].

Finally, FGFs have a role in supporting osteoblast differentiation, bone formation, and wound healing. Specifically, FGF-2 has an interesting role in tissue engineering. Momose et al. exploited this aspect and used FGF-2 in a collagen hydrogel scaffold and watched the effect on periodontal healing in the canine model over a 4-week period. They saw that the FGF-2 scaffolds caused cell and tissue ingrowth, along with vessel formation. They also observed alveolar bone regeneration from the scaffold [61]. This study suggests the ability of FGF-2 to regenerate both bone and vasculature in vivo. While growth factors are one of the main techniques being used to enhance scaffold functioning, other small molecules are also used. These include using deferoxamine, statins, dexamethasone, and platelet rich plasma combined with various types of 3D scaffolds. Yan et al. used a PCL scaffold combined with deferoxamine in vitro and in vivo. The scaffold was able to control the release of the deferoxamine, thereby enhancing angiogenesis and bone reconstruction. The deferoxamine was also able to increase vascular ingrowth and bone regeneration in the rat bone defect model [46]. Similarly, Yao et al. incorporated deferoxamine into a 3D nanoporous gelatin scaffold and observed its release. The deferoxamine was successfully released over 10 days in vitro. They observed increased angiogenic and osteogenic properties and found that the drug enhanced BMP-2 induced differentiation [62]. Another drug, simvastatin, has also been used with scaffolds to promote angiogenesis. Liyanage et al. loaded a poly (b amino ester) hydrogel scaffold with simvastatin and observed its release in vitro. The scaffold successfully released about 162 mg of simvastatin and stimulated preosteoblast activity over 20 days [63]. Encarnação et al. used PLGA and biphasic ceramic scaffold with added simvastatin and studied its release in vitro over 40 days. Simvastatin was released gradually over the full 40 days, without altering the chemical or mechanical properties of the scaffold [64]. Other studies have also used dexamethasone and studied its release from scaffolds. One study combined a silk fibronin/PLGA scaffold with dexamethasone and observed its diffusion in vitro over 21 days. The dexamethasone was quickly released from the 3D scaffold, which is favorable for proliferation and differentiation of BMSCs. The release of the drug was correlated with significant osteogenic differentiation [65]. Another study used dexamethasone with a porous poly (l-lactic acid) scaffold in vitro. Release tests showed that the drug had a controlled release for about a month. There were also no chemical changes in the drug with the addition to the scaffold. In vivo, the dexamethasone scaffold showed accelerated bone and blood vessel formation [66]. Finally, some studies have used platelet rich plasma (PRP) with scaffolds to enhance osteogenesis and angiogenesis. One such study used a platelet rich plasma hydrogel scaffold in vitro. The scaffolds showed good release of PRP, along with good osteoblast proliferation, viability, and attachment [67]. All of these studies and techniques suggest the wide range of additives that can enhance osteogenesis or angiogenesis. Growth factors remain the most commonly used to promote bone growth, but many drugs are also used to increase vascularization.

### 2.5. Effect of Cell Combinations

While GFs and other small molecules are useful in enhancing scaffold functioning, stem cells remain the one of the most common additions to 3D scaffolds. Stem cells provide the ability to differentiate into many types of cells and can be derived from a number of sources. The most common stem cells include bone marrow derived mesenchymal stem cells (BMSCs), synovial derived mesenchymal stem cells (SMSCs), adipose derived stem cells (ADSCs), dental pulp stem cells (DPSCs), and blood derived stem cells (BDSCs). Additionally, human umbilical vein endothelial cells (HUVECs) are also used to enhance scaffold functioning. BMSCs are the most common cell added to scaffolds and are removed from the bone marrow of a donor. They are also able to induce osteogenesis and angiogenesis when combined with scaffolds. Moreover, they are easy to proliferate and differentiate and have low immunogenicity. Sun et al. used BMSCs on a b-TCP scaffold and observed cell proliferation and differentiation in vitro. They found good proliferation and expansion of the stem cells, with potential for use in in vivo bone tissue engineering. Additionally, they saw an increase in activity of the BMSCs with their scaffold [68]. Another study used carboxymethyl chitosan/alginate 3D scaffold with BMSCs in an osteonecrosis of femoral head (ONFH) rabbit model over 12 weeks. The scaffold showed excretion of angiogenic and osteogenic factors, along with repair of the ONFH [69]. SMSCs are another type of cell used in conjunction with scaffolds to enhance osteogenesis. They are similar to BMSCs in that they have good proliferation and can differentiate into many types of cells. They have the added benefit of easy removal from a host and being multipotent. Lin et al. used SMSCs with a polyetherketoneketone scaffold and observed cell attachment, proliferation, and osteogenic potential. This was done in vitro and in vivo in a rabbit calvarial defect model. SMSCs attached to the scaffold and induced bone regeneration in vitro. Additionally, the stem cells regenerated double the amount of bone as the control in the rabbit model. This highlights their important role in osteogenesis and their potential use in osteonecrosis repair [70]. ADSCs can also be used to enhance angiogenesis in 3D scaffolds. They can also improve vascularization and give high stem cell yield. ADSCs have the added benefit of a simple and less painful removal procedure. Dębski et al. introduced ADSCs into 3D PCL scaffolds and observed the osteogenic and angiogenic effect in rats over 2 months. The scaffolds showed rapid vascular formation, suggesting their use in repairing ONFH. Additionally, the vessels formed from the stem cells and scaffolds were much denser than those without the stem cells [71]. DPSCs are also used in bone tissue engineering. They can differentiate into multiple cells types, have easy removal process, can be combined with many types of scaffolds, and can differentiate into osteoblasts. Jiménez et al. used DPSCs on a PLGA scaffold and evaluated the in vitro behavior over 30 days. They found that the scaffold with stem cells had a higher osteoblast differentiation capability and higher proliferation. This suggests a role for DPSCs in bone regeneration for the osteonecrosis model [72]. Moreover, Salgado et al. used DPSCs seeded on a collagen-nanohydroxyapatite/phosphoserine hydrogel 3D scaffold and observed its effects both in vitro and in vivo in immunocompromised mice. The cells showed high proliferation, viability, and osteogenic differentiation in vitro. In vivo, the DPSCs showed favorable differentiation into bone tissues with high potential for use in bone defect repair [73]. Another type of cell, BDSCs, can also be used for tissue repair. BDSCs have similar chondrogenic potential to BMSCs and can be obtained easily from a donor. Liu et al. used peripheral BDSCs seeded in a fibrin gel/PLGA microsphere scaffold to observe its bone regenerative qualities in vitro and in vivo in the rabbit model. The stem cells induced osteogenic differentiation and expressed elevated levels of osteogenic markers in vitro. In addition, new bone formation was observed in the rabbit model with an overall repair of the bone defect [74]. Another study developed a biphasic calcium phosphate bioceramic scaffold with rabbit BDSCs to enhance osteogenesis and vascularization in rabbit model bone defects. They found upregulated osteogenic and angiogenic gene markers along with biocompatibility of the scaffold in vitro. In the rabbit model, the scaffold and stem cells promoted new bone and vasculature growth, alluding to the cells’ potential ability to repair large bone defects [75]. Finally, HUVECs are a different type of cell used in tissue engineering. They have the ability to differentiate into cells that promote angiogenesis and even immune characters. Additionally, they can be easily isolated from fetal tissue. Chen et al. investigated the angiogenic and osteogenic capabilities of a nanomatrix scaffold with HUVECs in vitro. The scaffold and stem cells enhanced bone mineralization, alkaline phosphatase activity, and osteogenic gene expression. The scaffold also stimulated VEGF expression, leading to enhanced angiogenic capabilities. These results suggest the ability of HUVECs to both enhance bone repair and vascularization, potentially in the osteonecrosis model [76]. All of these types of cells represent promising methods to enhance osteogenesis and angiogenesis when combined with 3D scaffolds. In addition, their ability to regenerate bone and vasculature could be further exploited in order to devise a clinically translatable therapy for the treatment of osteonecrosis.

### 2.6. In Vivo Regeneration

In vitro studies are the ultimate test of scaffolds and their effectiveness for osteogenesis have the ability to show their osteogenic, angiogenic, mechanical performance, cell proliferation, and cell differentiation characteristics. Li et al. fabricated a porous gelatin/nano-lithium-hydroxyapatite/gelatin microsphere/erythropoietin composite scaffold and implanted it in the osteonecrotic femoral heads of rabbits after core decompression. They then evaluated the angiogenic, osteogenic, and defect repair ability of the scaffold. The scaffold showed enhanced new bone formation and improved femoral head defects. This highlights the scaffold’s role in repairing ONFH and the potential for its use in larger in vivo models [77]. Similarly, Zhu et al. used porous titanium/gelatin scaffolds to assess osteogenic properties in vivo. They used the rabbit bone femoral head defect model to assess in vivo properties. They saw a constant release of growth factors and no side effects from the scaffolds in the rabbit model. Additionally, micro-CT scanning showed a significantly higher bone volume compared to the control. 2 months after implantation, the scaffold had increased mature bone growth and was nearly identical to the unaffected femoral head. These results enforce the efficacy of this technique in repairing ONFH [78]. Luo et al. fabricated a novel calcium polyphosphate scaffold combined with Li and VEGF and assessed it in the ONFH rabbit model. They implanted the scaffolds via core decompression and observed the effects over 12 weeks. They saw improved osteogenesis and angiogenesis in the rabbit model with micro-CT displaying significantly better bone volume and density compared to the control. There was also enhanced new bone formation and expression osteogenic factors [79]. Another study created a silk fibroin/hydroxypropyl methylcellulose scaffold and used it in the rabbit femoral head core decompression model over 2 months. The scaffold showed excellent mechanical performance in vivo with no inflammatory response. Moreover, micro-CT showed that the scaffolds induced significantly more bone formation [80]. Wang et al. developed a PCL/Cervi cornus Colla deproteinized bone scaffold and implanted it in ONFH induced rats. The researchers then observed the reparative effect of the scaffold in the rats. X-ray imagery showed significant repair of ONFH from the scaffold. Their femoral heads had less destruction of chondrocytes along with limited necrosis. In addition, the rats showed no signs of an immune response to the scaffold [81]. Zhu et al. used a novel PLLA/PLGA/PCL scaffold seeded with BMP and combined with low intensity pulsed ultrasound (LIPUS) to observe its effect in repairing ONFH in the rat model. The combination of the scaffold and LIPUS showed repair of ONFH and enhanced load bearing capability. Micro-CT scans showed rats with the scaffold had significantly higher bone density, bone volume, and trabecular thickness. The scaffolds also elevated bone formation rates and bone angiogenesis. Overall, the scaffold showed very good ability to reverse ONFH and contribute to additional bone formation [82]. Mou et al. investigated the osteoinductive effect of a copper doped calcium deficient hydroxyapatite/multi (amino acid) copolymer scaffold in the rabbit model over 12 weeks. Significant new bone formation was observed by X-ray imaging with the scaffold. There was also more vascularization around the scaffolds with enhanced osteogenesis [83]. Moreover, Kang et al. developed a strontium doped calcium polyphosphate with autologous bone marrow mononuclear cells and implanted them into the rabbit model of ONFH to observe over a 12 week period. X-ray observation showed that the scaffold promoted healing of the FH defect. Additionally, there was significant bone growth in and around the scaffold site. There was also no inflammatory reaction from the scaffolds after 12 weeks [84]. Maruyama et al. used functionally graded PCL/b-TCP scaffolds with BM mononuclear cells to observe its effect in repairing ONFH in the rabbit model. Micro-CT analysis showed increased bone ingrowth from the scaffolds and increased bone volume. Moreover, the scaffolds showed a decrease in necrotic bone region area compared to the control [85].

## 3. Tissue Engineering/Regenerative Therapies

**Figure 4:**
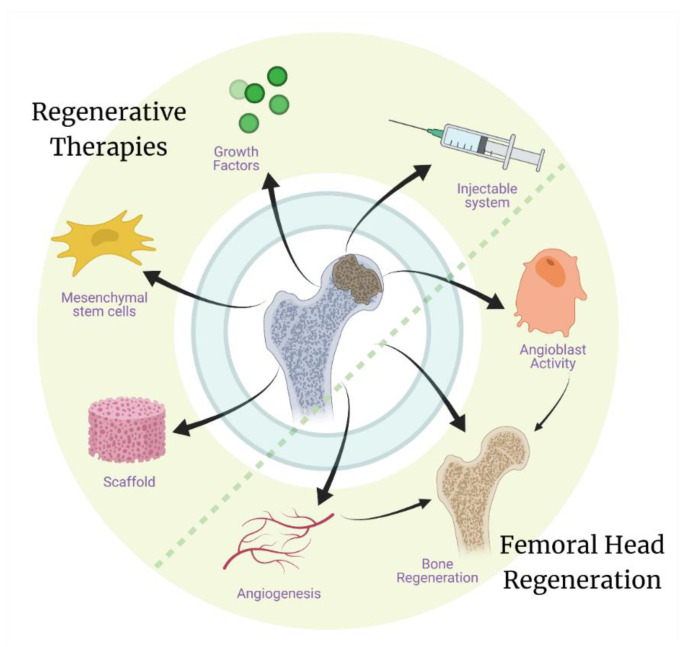
Regenerative therapies, including use of grafts/scaffolds with MSCs and growth factors, have shown promise in studies investigating the regeneration of the necrotic femoral head. While the development of a therapeutic system that can be delivered through a minimally invasive surgery will evade the surgical approach completely and can potentially help in both the regeneration of vasculature and the bone tissue.

Many recent studies have sought to define and modify the properties of tissue engineered material systems in order to develop better regenerative therapies for AVN. Increasing knowledge about the pathobiology of the disease will further aid regenerative scientists to devise improved tissue engineering strategies [86]. The technologies most utilized in tissue engineering are cellular therapies (usually with bone marrow-derived mesenchymal stem cells-BMSCs), growth factor therapies, metallic implants, and 3D bioprinting and nano printing for ceramic/polymeric scaffolds (Figure 4). Most of these technologies are in experimental phases and thus have their specific advantages and disadvantages, which are being further studied to improve these strategies.

### 3.1. Cellular Therapies

Cellular therapies are seen to be mostly effective for early stage 2 AVN [87]. They mainly include the use of MSCs as they are responsible for the regeneration of bone and cartilage cells in the body. Adipose derived stem cells (ADSCs) have also been exploited in some studies to devise regenerative therapies for AVN [88]. MSCs can be derived from bone-marrow through aspirates, culture, or concentrates (Andriolo et al., 2018). They can also be derived from adipose tissue or umbilical cord (Andriolo et al., 2018) and can be injected intra-arterially or directly into the necrotic area. MSCs have been shown to repair and regenerate bone due to their multipotentiality, paracrine signaling molecules, and “ability to home to the injured tissue” [89]. The advantage of cellular therapies are that they are potentially less invasive than surgical treatment [90]. MSCs have been observed to be associated with the regeneration of bone tissue as well as the initiation of re-vascularization of the necrotic tissue in AVN. MSCs also regulate the process of both bone formation and resorption through the secretion of different cytokines like IL-1β, IL-6, IL-11, OPG; growth factors such as PDGF, TGF-β, LIF, FGF-2, M-CSF; chemokines like RANKL; and other molecules, including DKK-1, PGE-2 and Wnt [91]. MSCs regulate the process of osteoclast formation and their inhibition through the NF-κB pathway, where RANK is involved in the promotion while OPG is involved in the inhibition of the osteoclasts [92].

A randomized and controlled study conducted by Li et al. in 2013 explored the effectiveness of allogeneic MSCs intravenously transplanted into rabbit models (sample size of 45). The repair progress was monitored, and it was found that there was bone regeneration of the necrotic bone in the femoral head, and vascularization was promoted. This showed promise as an effective minimally invasive treatment [93]. Pak et al. conducted two case reports to explore the effectiveness of adipose-tissue derived stem cells and platelet-rich plasma in regenerating bone. It was found that medullary bone-like tissue was regenerated in the area of necrosis of the femoral head for the two patients [94].

Stem cell therapies can be used in conjunction with medication. Li et al. in 2010 used BMSCs along with pravastatin (a statin medication) to treat early stage AVN of the femoral head in 32 patients’ (49) hips. The results showed that pain lessened, and function improved. New vasculature was formed in 21 hips. This study provided some evidence for the effectiveness of cellular therapies combined with medication; however, the study was not randomized or controlled [95]. Another study by Li et al. investigated the effectiveness of MSCs with a lithium chloride treatment. They injected 48 rabbits with differing concentrations of lithium chloride in a randomized controlled experiment and found that trabecular bone density and mass of the femoral head was restored with the optimal lithium chloride concentration (10 mmol/L). Vascularization was not observed in this study [96]. Wu et al. conducted a randomized and controlled study with 72 male rabbits using a combination of MSCs and Danshen, a Chinese herbal medicine commonly known as red sage that is used as an anti-cancer agent. Re-ossification and vascularization was observed in the necrotic area of the femoral head [97].

Recently, genetic engineering has emerged to improve the effectiveness of MSCs in bone regeneration of femoral head necrosis. MSCs transfected with genes for enhanced production of growth factors, such as VEGF, FGF, and BMP, can improve the regeneration capacity of these cells by initiating a heightened signaling response for cellular recruitment and initiation of anabolic activities, including bone formation and vascularization. It was demonstrated in a rabbit model that FGF-2-transfected MSCs in a xenogeneic antigen-cancellous bone (XACB) scaffold can improve bone regeneration. It was found that TNF-α expression was inhibited by increased FGF-2 expression, which might have triggered the improved bone regeneration response in the steroid-induced osteonecrosis rabbit model [98]. Another study used bone MSCs transfected with both VEGF and BMP-6 in conjugation with a poly lactide-co-glycolide (PLAGA) hydrogel that were implanted subcutaneously in nude mice. The tissue demonstrated increased bone formation and angiogenesis after 4 weeks, providing evidence for the potential of these cells in the treatment of AVN [99]. BMP-2 and basic-FGF were expressed in bone marrow stem cells (BMSCs), through an adenovirus mediated expression system in conjugation with demineralized bone matrix (DBM) in a canine model. The approach was associated with increased regenerated bone with increased vascularization and enhanced bending and compressive strength of bone compared to controls in the AVN model [100].

MSCs have some disadvantages linked to their low yield and painful extraction process, which can involve surgical complications. ADSCs, which can be rather easily isolated and have a significantly greater yield than MSCs, have thus been explored for the regeneration of bone in AVN. It has been demonstrated that osteogenically induced ADSCs can induce bone regeneration in a rabbit model [101]. A clinical study demonstrated the use of ADSCs in two patients where autologous ADSCs were injected into the affected hips and the patients were examined after 3 months. The study demonstrated improved Harris score and with MRI showing increased regeneration [94]. Other stem cells, such as dental-pulp stem cells (DPSC), synovial-derived mesenchymal stem cells (SDMSC), blood-derived mesenchymal stem cells (BDMSC), and umbilical cord-derived mesenchymal stem cells (UCDMSC), have also been explored for bone regeneration in AVN [37]. Thus, other types of easily available stem cells that can differentiate towards the osteogenic lineage can also be explored for the treatment of AVN.

### 3.2. Growth Factor Therapies

Growth factors can be used to promote stem cell differentiation and vasculogenesis. There are many different growth factors that promote osteogenesis and bone healing. Some include bone morphogenetic protein (BMP), vascular endothelial growth factor (VEGF), hepatocyte growth factors (HGF), and platelet derived growth factors (Table 1). Growth factors in general help MSCs differentiate and proliferate into osteoblasts and chondroblasts [102]. Advantages of using growth factors in treatment for AVN of the femoral head include avoidance of additional surgical interventions as most of these can be injected while they can also be administered in conjugation with surgical treatments and tissue engineered grafts/scaffolds.

BMPs stimulate mesenchymal progenitor cells to form bone and cartilage [102]. This would be of obvious benefit in cases of AVN of the femoral head. Subsets 2, 6, and 7 of BMPs have been shown to be particularly effective [102]. BMPs are often used in conjunction with VEGF, an angiogenic growth factor that helps promote vascularization [102]. Ma et al. used BMP-2 and VEGF-165 with BMSCs in 36 rabbits with induced AVN in the right femoral head. The randomized groups were single core decompression, core decompression and BMSCs, and core decompression with BMP-2/VEGF-165 BMSCs. The results showed that bone repair and vasculogenesis was significantly greater in the latter group, further demonstrating the effectiveness of these two growth factors in differentiation of BMSCs and angiogenesis [116]. In an in vitro study carried out by Liao et al. in 2018, BMSCs (2 × 10^5^/mL) from rats were seeded onto plates and transfected with BMP-6 and VEGF growth factors. They were then seeded onto PLAGA (poly lactide-co-glycolide) scaffolds, and the angiogenesis and bone regeneration were observed in vivo. The results showed that the addition of growth factors were much more effective in promotion of bone and vascular growth [99]. Wang et al. used Colla Cornus Cervi (CCC) and BMP-7 transfected BMSCs in AVN induced rat models. CCC is deer antler glue, and it is said to have osteogenic potential. The results showed that there was extensive proliferation and osteogenesis in the models with the experimental treatment [117].

Hepatocyte growth factor (HGF) is an endothelial growth factor, and it can stimulate vasculogenesis similar to VEGF [103]. It has been found to be a more potent cell differentiation promoter than VEGF [103]. HGF at high concentration was found to be very effective in osteogenic differentiation of MSCs and tissue repair in rabbit models [115]. In a study carried out by Wen et al. in 2014, HGF combined with fibrin glue (material that supports cell differentiation) was transfected into rabbit derived MSCs. These cells were observed in vivo in 30 rabbit models (randomized and controlled groups were used). It was found that HGF significantly promoted cell differentiation and vasculogenesis, and fibrin glue supported differentiation and regeneration of femoral head necrosis [118].

Other growth factors utilized include platelet derived growth factors. Platelet-rich plasma derived growth factors (PRP-GFC) is an autologous source of many different growth factors and have been associated with cartilage regeneration [119]. In a study conducted by Nandeesh et al. in 2016, 48 patients were treated with PRP-GFC and BMSCs, and it was found that the patients had improved motor function and cartilage regrowth. Thus, growth factor therapies are another potential tool for bone regeneration and vascularization. However, all of these approaches fail to initially stabilize the biomechanics of the collapsing femoral head, which might not allow sufficient time for the tissue heal because of recurring damage. Thus, conjugating growth factors with injectable material systems which support the collapsing cartilage can potentially develop a therapeutic system with both the advantages of mechanical support to the femoral head and regeneration simultaneously.

### 3.3. Metallic Implants

Another treatment method for AVN of the femoral head is metallic implants. Metallic implants provide the mechanical support that is missing in non-structural bone grafting, leading it to be a candidate in helping with pre- and even post-collapse lesions in AVN of the femoral head. There are several metals being used in these implants, which have been chosen based on various properties, such as biocompatibility, strength, and elasticity. Two of the most common metals used are titanium and tantalum.

#### 3.3.1. Porous Titanium Rods

Titanium implants have been used in orthopedic surgeries for many years due to their optimal characteristics. Titanium is strong, rigid, and has good mechanical properties for use in joint and bone repair. Additionally, it has good biocompatibility for use in patients. Porosity can also be introduced into these rods to enhance similarity to human bone tissue. Therefore, porous titanium rods offer a promising option for repair of ONFH. Zhang et al. developed a 3D printed porous titanium metal trabecular bone reconstruction system and evaluated its effectiveness in repairing ONFH in 30 patients over 24 months. They observed significant Harris score increases and visual analogue scale decreases in very short term follow-up at 12 months after implantation. Additionally, 100% of ARCO IIA, 70% of ARCO IIB, and 0% of ARCO IIC hips showed postoperative improvement. They concluded that the titanium implants were most effective for early treatment of ONFH in ARCO IIA and IIB hips. However, the implants were not beneficial for ARCO IIC patients [120]. Wang et al. developed a porous titanium alloy rod with a diamond crystal lattice and implanted them in vivo in ONFH sheep for 6 months. The rods showed adequate osteoconduction, along with good new bone growth. Moreover, the titanium implants showed higher bone volume on micro-CT scans, enhanced bone ingrowth, and increased mechanical properties. These rods showed promising ability to treat early onset ONFH [121]. Another study used a 3D porous titanium scaffold in the ONFH dog model and evaluated its treatment ability over 12 weeks. Micro-CT showed moderate repair of ONFH, but still displayed some FH collapse. They also observed proliferation and differentiation of osteoblasts with the titanium scaffold and inhibition of osteoclast proliferation. The titanium groups also showed upregulated bone regeneration markers, such as BMP-2, VEGF, b-FGF, and RUNX2. The titanium treatment overall showed good repair of ONFH in the canine model [43]. Wang et al. observed biogenic trabecular porous titanium rods with a lamellar configuration in early ONFH sheep models. The rods were also combined with core decompression and analyzed after 6 months. The rods displayed better bone ingrowth, stronger mechanical properties, and increased bone volume. This treatment also shows a possible method to repair early ONFH [122]. Similarly, Zhang et al. developed a novel porous Ti6Al4V (titanium) scaffold with diamond lattice pore structure and observed its osteogenic properties in an ON canine model over 6 months. The scaffolds showed promising mechanical properties and good biocompatibility in vivo. In addition, the scaffolds showed increased bone ingrowth and mineral density. This study demonstrates how scaffolds can be successfully manufactured to match the mechanical properties of bone [123]. Zhu et al. observed the reparative effect of 3D printed porous titanium rods implanted in gelatin in the ONFH rabbit model over 2 months. The scaffolds showed accelerated bone regrowth in vivo on micro-CT. Moreover, trabecular bone regenerated by the scaffold was nearly identical to unaffected trabecular bone. The bone volume in the scaffold group was also significantly higher than the control [78]. Finally, Wang et al. developed a custom 3D printed porous titanium sleeve implant to repair a femoral head defect in a 73-year-old ONFH patient. The patient had no adverse symptoms and displayed a Harris score of 91, 2 years after implantation. The implant also showed good bone ingrowth and suggests its ability to repair FH defects [124]. Overall, porous titanium implants have favorable characteristics for use in early ONFH repair in vivo. However, they fail to repair ONFH and support the implant region without the use of other materials. Therefore, they are not a suitable treatment alone and are not commonly utilized for the clinical treatment of AVN.

#### 3.3.2. Porous Tantalum Rods

Porous tantalum rods are the most widely used metallic implant in treatment of AVN of the femoral head. Porous tantalum is praised for its biocompatibility and excellent mechanical properties. Notably, its modulus of elasticity and compression strength values fall between those of cancellous and cortical bone; with similar properties of human bone tissues, it is the medium of choice for treatments involving metallic implants [125]. The honeycomb structure resembles cancellous bone, which is also helpful in support and bone growth. Tsao el al. was first to conduct a multiple-center clinical study investigating the use of porous tantalum rods to treat AVN of the femoral head in 2005. A total of 113 rods were surgically implanted in 98 patients with either Steinberg stage-I or stage-II AVN, which resulted in overall improved HHS in patients, improving from 63 preoperatively to 84 after 4 years [126]. In addition, the survival rate of the femoral head in patients after 48 months was 72.5%, indicating positive results in treatment even after extended follow-up. These results showed promise in the use of porous tantalum rods as a viable treatment option for early-stage AVN of the femoral head, which led to experimentation to improve the efficacy of them even further. In this way, favorable results have often been accomplished through pairing the porous tantalum implant with core decompression, bone grafting, or various populations of stem cells. One such study involved the pairing of the implant with core decompression, in which 52 patients with 58 hips affected by AVN of the femoral head received the two treatments together and were followed-up at a mean of 24 months. The results showed that only nine hips progressed to require THA, which all began in stage-II or stage-III AVN. Survival rates decreased substantially at each 12-month interval but remained at 92% at 48 months in those without chronic systemic diseases [127]. This study showed promising results in treatment of AVN in the absence of chronic systemic diseases, especially in early-stage AVN, with porous tantalum rods inserted during core decompression surgery. In addition, the study praised the technique for being minimally invasive, requiring less surgery time than other treatments, and having no donor-site morbidity. In another study, porous tantalum rods were combined with autologous bone grafting and bone marrow cells to treat 49 patients with 58 hips in Steinberg stage-II or stage-III AVN, and patients were followed up after 5 years. Results indicated a 93.1% success rate based on conversion to THA and an 87.9% success rate based on disease progression as well as significantly improved Merle d’Aubigne scores, indicating that the combined treatment method was effective in slowing the advancement of AVN [128]. However, many studies have also found failed outcomes using these implants. In one study, 15 failed porous tantalum implants out of 113 implanted to treat Steinberg stage-II AVN of the femoral head were analyzed. The study found residual necrosis in 14 of the 15 patients, fracture of the subchondral bone in all patients, and collapse of the femoral head in 9 patients [129]. Bone ingrowth was found in 13 of the 15 specimens, but the extent of bone growth was insufficient for proper repair and healing to take place, leading to these unfavorable results. There are several other studies that have come to similar disheartening results, but no conclusive failure mechanism for porous tantalum rods in AVN of the femoral head has been decided. Mixed results in efficacy of this treatment option indicate the need for further clinical trials and experimentation involving the porous tantalum rods alone and in combination with other methods that may improve outcomes. Further, most studies examine AVN in patients of middle to advanced age and may not be translatable to highly active young patients. 

### 3.4. Ceramic/Polymeric Scaffolds

Ceramic and polymeric scaffolds can offer the advantage of initial biomechanical support to the collapsing femoral head and can also be used as a tissue engineering treatment therapy for AVN of the femoral head. They can mimic the environment in which the cells grow and provide a place for angiogenesis and differentiation. Liao and co-workers recently reported an in vitro study with BMSCs and BMP-6/VEGF growth factors seeded onto 3D printed PLAGA (poly lactide-co-glycolide) scaffolds [38]. Angiogenesis and bone regeneration were observed when implanted in vivo. The 3D printed scaffold and growth factors provided a microenvironment that supported cell differentiation. In another study carried out by Wang et al., BMSCs were seeded onto nano-hydroxyapatite/collage I/poly-L-lactic acid (nHAC/PLA) scaffolds. When studied in vivo, angiogenesis was observed near the necrotic bone of the femoral head, and new osteoid tissue was formed. Those results provided support of the use of a polymeric scaffold as an effective treatment for AVN of femoral head [130]. Another study carried out by Luo et al. showed BMSCs seeded onto lithium calcium polyphosphate (LiCPP) scaffold containing many other growth factors to be effective in treating AVN because of its osteogenic and angiogenic activity [79]. 3D printing has been used recently in some studies to design scaffolds for treatment of AVN. A functionally graded scaffold was 3D printed and composed of polycaprolactone (PCL) and β-tricalcium phosphate (β-TCP) [44]. The printed scaffolds had a spatially controlled porous profile as well as desired degradation rates and mechanical strength properties. The construct had less porosity at the ends while having increased porosity in the middle portion, consistent with the desired mechanical properties needed for the tissue. The scaffolds were implanted in a drilled cavity in rabbit femoral head and neck. The samples were examined after 8 weeks of implantation and showed high mineralized in micro-CT and no-bone formation in histological studies, with an increased scaffold resorption on both the ends of the scaffolds versus controls. Another study demonstrated the fabrication of a β-TCP scaffold modified with a bone marrow-derived mesenchymal stem cell (BMSC) affinity peptide through an adsorption and freeze-drying method [131]. In a rabbit model of early AVN, the scaffolds were implanted after core decompression. It was demonstrated that the scaffolds had a good affinity towards BMSCs. The scaffolds demonstrated higher bone regeneration than the controls and are thus a step forward from results of current surgical interventions. Lai et al. demonstrated the fabrication of a poly (lactic-co-glycolic acid)/β-calcium phosphate/icariin (PLGA/TCP/Icariin, PTI) scaffold. Icariin is a phytomolecule that can facilitate bone regeneration [132]. In this study of a rabbit model of AVN, these bio-functional scaffolds were implanted after core decompression. The scaffolds were found to enhance the mechanical properties of the tissue and resulted in enhanced angiogenesis and bone regeneration. Another interesting strategy was demonstrated by Peng and co-workers where they fabricated biphasic calcium phosphate (BCP) ceramic scaffolds based on micro-CT images through a 3D gel-lamination technique mimicking the cancellous bone microarchitecture of femoral heads [133]. Bone marrow-derived mesenchymal stem cells (BMSCs) were seeded on these scaffolds and then implanted in a canine bone defect in the femoral head. After 30 weeks of implantation, significantly enhanced bone regeneration was observed in the BCP scaffolds, with an increase in the mechanical properties of the regenerated site. This study further supports the utility of tissue engineering strategies, not only in regenerating the tissue, but also in providing and maintaining support to the collapsing femoral head to reduce the progression of osteoarthritis after AVN.

Studies have also utilized metal ions for inducing bone regeneration for AVN treatment. A study demonstrated the fabrication of poly (lactide-co-glycolide) (PLGA), β-tricalcium phosphate (β-TCP) scaffolds with magnesium ions through low temperature rapid prototyping (LT-RP) technology [39]. These 3D-printed scaffolds in a rabbit model of AVN demonstrated neo-angiogenesis and increased blood perfusion after 4 weeks by dynamic contrast-enhanced MRI and micro-CT based angiography. At 8 weeks, newly formed blood vessels were observed, while at 12 weeks enhanced bone formation was observed with increased mechanical properties of the tissue. After 12 weeks, the scaffolds did not illicit any increase in the serum Mg levels. These scaffolds demonstrate that metallic ions that can be harnessed through degradable polymeric scaffolds with ceramic components to improve regeneration of bone in AVN. In another study, lithium ions were incorporated into scaffolds to enhance bone regeneration [77]. Porous gelatin/nano-lithium-hydroxyapatite/gelatin microsphere/rhEPO (Li-nHA/GMs/rhEPO) were fabricated with the intention of activating Wnt signaling through lithium ions, while erythrogenin (EPO) upregulated to the HIF-1/VEGF pathway for bone and vascular regeneration. The scaffolds demonstrated increased viability of glucocorticoid-treated BMMSCs and vascular endothelial cells and increased the expression of osteogenic and angiogenic factors in vitro. These scaffolds were implanted in a rabbit model of AVN after core decompression procedure, which demonstrated enhanced bone formation and angiogenesis, with enhanced mechanical properties. Though these engineered tissues are currently being used in conjugation with current surgical procedures for the treatment of AVN, further development may result in their future use as isolated procedures with equivalent or better clinical efficacy in the treatment of AVN. 

## 4. Future Perspective

Overall, currently available surgical treatments for AVN are able to partially manage the pathology and are not successful in a large number of cases. This is also dependent on the timely diagnosis of the disease, which plays an important role in the success of surgical strategy. For example, core decompression technique works well in the early stages of AVN. While, the same technique is not very effective in the later stages, as the necrotic area has already increased and the collapse of the femoral head can’t be avoided. Further, many of these early stage cases of AVN may resolve or remodel independent of treatment with outcomes similar to those of surgically treated patients further complicating the utility of current approaches such as core decompression. Clinicians have been trying to combine surgical approaches with autografts, vascular implants and metallic implants; but these hybrid treatments are also partially successful as they are not able to initiate the regeneration of the bone tissue to replace the necrotic core and help it re-vascularize. While metallic implants provide excellent mechanical support but are totally inert towards regeneration of the tissue and also take up the space in which the neo-tissue can grow. Further, there is often a mismatch in the properties of the implants leading to the undesired mechanical destruction of the tissue surrounding the implant. Thus, in order to devise a successful clinical treatment strategy for management of AVN, we need a material system that can provide the desired mechanical support in the initial stages to support the collapsing femoral head and at the same time, guide the recruitment, differentiation, and regeneration of bone tissue and supporting vasculature in the necrotic area. Biomaterial approaches represent a viable solution for this as they can be modified to include both osteogenic and vasculogenic growth factors to initiate regeneration, while their mechanical properties can be modulated to support the collapsing femoral head. The degradability profile of biomaterials can also be regulated to make space for the regenerating bone tissue so that the implanted material is resorbed while the neo-tissue grows to support the femoral head.

To date, available biomaterial systems are able to initiate regeneration in the necrotic tissue, but are typically not able to support the biomechanical loads to which the femur head is exposed. Thus, development of biomaterials that are mechanically strong and can stabilize the femoral head are required for AVN treatment. Implantation or placement of such a system while preserving as much healthy tissue as possible and limiting further mechanical destabilization of articular cartilage is paramount. Thus, research towards injectable systems that can be delivered through a minimal invasive surgery needs to be focused on. As previously discussed, the most important aspect in developing such a system is that they need to be mechanically strong while being injectable, thus material systems that can regenerate their structural network or create it after being injected will be the ideal solutions. These systems can potentially carry biochemical cues for the regeneration of bone as well as vasculature, leading to regeneration of the necrotic femoral head. Such strategies can potentially provide us with clinically translational therapies that can help in preserving the hip with minimal disruption to healthy tissue or the cartilage of the articular surface.

## Figures and Tables

**Table 1:** Use of growth factors for the regeneration of the bone and vasculature of the necrotic femoral heads have been practiced clinically. They can be injected or delivered through overexpression by genetically transfected stem cells.

Growth Factor	Associated Cells	Delivery Strategy	Regeneration Results	Reference
Hepatocyte growth factor (HGF)	BMSCs	HGF transgenic BMSCs transplanted using core decompression (CD) with fibrinogen drug delivery mixture (FG)	Formation of new capillaries on bone plates of the trabeculae. Bone marrow rich in hematopoietic tissue.	[103]
Granulocyte colony stimulating factor (G-CSF) and stem cell factor (SCF)		G-CSF and SCF injected subcutaneously for 5 days mobilizing BMSCs	Increase in osteocalcin protein expression. Vessel formation was 3.3 fold greater & vessel density was 2.6 fold greater than the control.	[104]
Vascular endothelial growth factor (VEGF)		Plasmid encoding VEGF immobilized on a cartilage carrier into the necrotic area of the femoral head	Increase in bone formation after 8 weeks.	[105]
Bone morphogenetic protein (BMP-2)	BMSCs	Modified BMSCs loaded onto *β*-TCP cylinder and implanted into core tract from CD	Increased amounts of new bone and higher maximum compressive strength and bone density.	[106]
BMP-2 and BMP-14		BMP-laden collagen scaffolds transplanted following CD	BMP-14 loaded scaffolds improved bony remodeling of the necrotic area	[107]
VEGF		VEGF injected continuously or through osmotic micropump	Reversal of osteonecrosis.	[108]
Recombinant human fibroblast growth factor (rhFGF)-2		rhFFGF-2 impregnated gelatin hydrogel administered locally	Increased Harris hip score. Reduction in pain level.	[109]
VEGF		Deproteinized bone (DPB) with recombinant plasmid pcDNA3.1-hVEGF165 was implanted into the drilled tunnel of necrotic femoral head	Increased bone formation and capillary vessel regeneration	[110]
VEGF	BMSCs	Transgenic autologous BMSCs implanted following CD	Enhanced bone reconstruction and blood vessel regeneration.	[111]
rhBMP-2		Cavity was made using the light bulb technique and autologous cancellous bone combination of rhBMP-2 filled the cavity	May be effective in avoiding future THR in younger patients and improve the speed of bone repair (Lack of statistical significance)	[112]
rhBMP-7		Fibular graft harvested from femoral neck, sprinkled with rhBMP-7 and implanted in the tunnel	Increased Harris hip score. Decrease in pain. Retention in the sphericity of the femoral head.	[113]
BMP-2		Percutaneous intraosseous injection of BMP-2 and ibandronate	Decreased femoral head deformity and increased bone formation.	[114]
HGF	MSCs	Transplantation of HGF-transgenic MSCs through CD tunnel	Increased the number of MSCs and osteogenic differentiation of MSCs.	[115]
